# Building a Cost-Efficient High-Pressure Cell for Online High-Field NMR and MRI Using Standard Static Probe Heads: An In Situ Demonstration on Clathrate Hydrate Formation

**DOI:** 10.1021/acs.analchem.3c03050

**Published:** 2023-11-06

**Authors:** Maarten Houlleberghs, Shannon Helsper, Dirk Dom, Thierry Dubroca, Bianca Trociewitz, Robert W. Schurko, Sambhu Radhakrishnan, Eric Breynaert

**Affiliations:** † NMR/X-Ray Platform for Convergence Research (NMRCoRe), KU Leuven, Leuven 3001, Belgium; ‡ Centre for Surface Chemistry and Catalysis: Characterization and Application Team (COK-kat), Leuven 3001, Belgium; § Biomedical MRI, Department of Imaging & Pathology, KU Leuven, Leuven B-3000, Belgium; ∥ National High Magnetic Field Laboratory, Tallahassee, Florida 32310, United States; ⊥ Department of Chemistry and Biochemistry, Florida State University, Tallahassee, Florida 32306, United States; # Centre for Molecular Water Science (CMWS), 22607 Hamburg, Germany

## Abstract

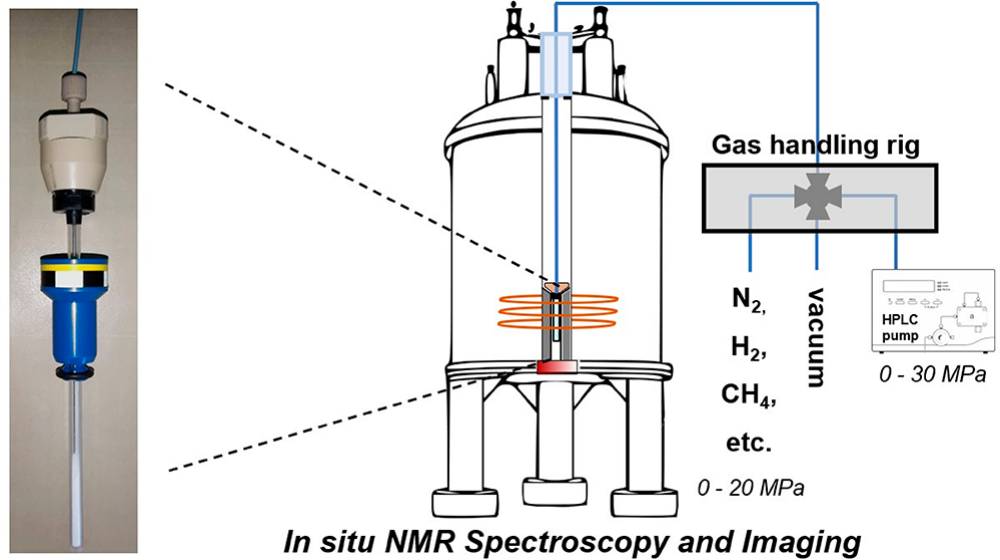

High-pressure nuclear magnetic resonance (NMR) spectroscopy finds remarkable applications in catalysis, protein biochemistry and biophysics, analytical chemistry, material science, energy, and environmental control but requires expensive probe heads and/or sample cells. This contribution describes the design, construction, and testing of a low-cost 5 mm NMR tube suitable for high-pressure NMR measurements of up to 30 MPa. The sample cell comprises a standard, 5 mm single-crystal sapphire tube that has been fitted to a section of a relatively inexpensive polyether ether ketone (PEEK) HPLC column. PEEK HPLC tubing and connectors enable integration with a gas rig or a standard HPLC pump located outside the stray field of the magnet. The cell is compatible with any 5 mm static NMR probe head, exhibits almost zero background in NMR experiments, and is compatible with any liquid, gas, temperature, or pressure range encountered in HPLC experimentation. A specifically designed transport case enables the safe handling of the pressurized tube outside the probe head. The performance of the setup was evaluated using *in situ* high-field NMR spectroscopy and MRI performed during the formation of bulk and nanoconfined clathrate hydrates occluding methane, ethane, and hydrogen.

High-pressure nuclear magnetic resonance (NMR) spectroscopy was originally developed to study the mechanisms, kinetics, and thermodynamics of liquid-phase homogeneous catalytic reactions, often involving systems developing autogenous pressure or being pressurized by gases.^1,2^ Over the past decades, the technique has found remarkable applications in protein biochemistry and biophysics, analytical chemistry, material science, energy, and environmental control.^3−8^ Also in magnetic resonance imaging (MRI), high-pressure magnetic resonance experiments have proven their value, enabling, for example, the visualization of the formation of mixed CO_2_–CH_4_ clathrate hydrates in porous media.^9,10^

Clathrate hydrates, referred to as “hydrates” hereafter, are ice-like inclusion compounds exclusively consisting of hydrogen-bonded water molecules that typically form by contacting water and methane under high-pressure and low-temperature conditions (up to 10 MPa at 274.2 K). Clathrate hydrates comprise different structural cages which are large enough to occlude nonpolar guest molecules, such as CO_2_, CH_4_, and C_2_H_6_.^8,9^ The storage capacity of these materials is remarkable: 1 m^3^ of clathrate hydrate can contain 160 m^3^ of gas at standard temperature and pressure (273.15 K and 0.1 MPa).^11^ This unique property has ignited the interest of the scientific community and prompted extensive research into the application of clathrate hydrates as potential storage technologies, focusing mainly at overcoming the significant heat and mass transfer limitations plaguing their scale up.^12^ While commercial high-pressure NMR sample cells can easily reach the required pressure conditions for clathrate studies, low-cost, versatile alternatives are a valuable addition, as they can render *in situ* high-pressure NMR experimentation more accessible, paving the way for new discoveries and insights in research fields relying on pressure ranges extending up to 30 MPa.

In general, MR experiments at high pressures are enabled by pressurizing the sample holder or the complete probe head (including the sample holder).^4^ The latter is referred to as the Jonas method and enables experiments in the GPa range.^13^ The downside is that it requires special high-pressure, nonmagnetic, metallic probe heads. The design of such probe heads is problematic. It requires reliable, low-impedance radiofrequency feedthroughs to be constructed through thick metal parts of the high-pressure probe head, and the construction should also fit in the bore of the magnet without perturbing the field homogeneity.^14^ Pressurizing only the sample holder is known as the Yamada glass cell method. It was originally developed using glass capillaries as high-pressure cells, a technique that has been successful in synchrotron experimentation.^15^ While compatible with most commercial NMR probe heads, the glass capillaries (typically borosilicate or quartz) used in this approach are fragile and require thick walls to ensure pressure resistance,^16^ limiting sample volume and thus sensitivity. For *in situ* experiments requiring pressure changes up to ∼400 MPa, alternative designs involving tubes composed of sapphire single crystals, plastics, ceramics, and other composites have been used.^4,17,18^ Typically, these tubes are mounted in expensive, nonmagnetic, metallic manifolds (often titanium based) to connect the sample environment to a gas handling rig without impacting the magnetic field homogeneity.^4^ Zirconia-based high-pressure NMR cells with titanium manifolds withstanding pressures up to 400 MPa, for example, are commercialized by Daedalus Innovations,^19^ but they cost upward of $10,000. Alternative NMR- and MR-compatible flow-through sample cells at high pressure have also been designed to study hydrate formation in sediments and at subseafloor conditions, but their complexity comes with its own challenges.^20,21^

To overcome the complexity and high costs associated with high-pressure NMR, a low-cost sample environment suitable for NMR measurements of up to 30 MPa was developed. The performance of this new high-pressure sample environment was evaluated in high-field *in situ* studies monitoring the formation of clathrate hydrates of methane and ethane with ^13^C NMR and ^1^H MR spectroscopy and imaging.

## Tube Assembly and Testing

### Tube Assembly

**Figure 1:**
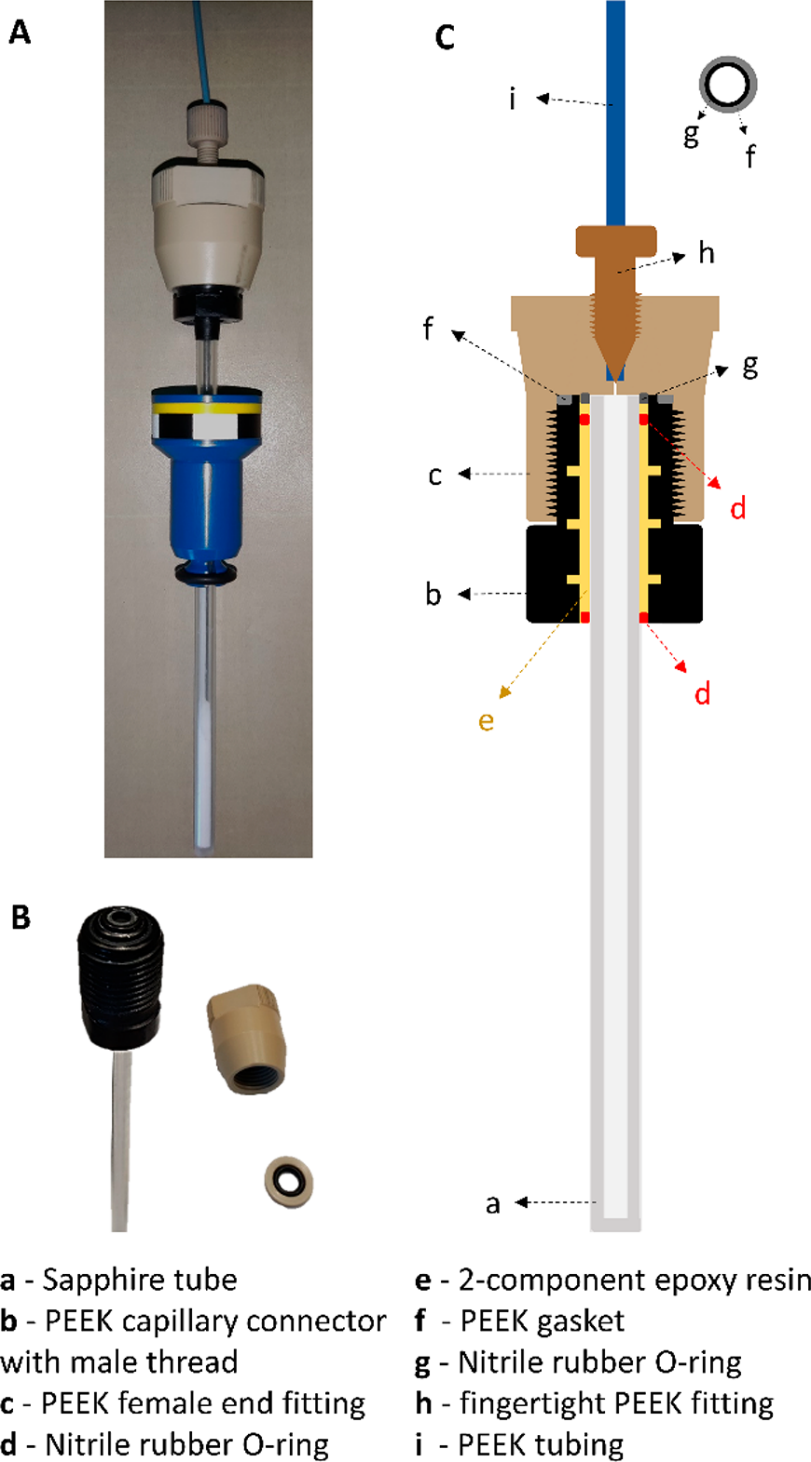
Photographs (A, B) and schematic representation (C) of the different components of the high-pressure NMR sample cell based on a 5 mm sapphire tube retrofitted with a capillary connector with a male thread derived from a PEEK HPLC column.

A 5 mm sapphire tube (Al_2_O_3_ single crystal; SP Wilmad-LabGlass; WG-507-7 series) with outer diameter (OD) 4.92 ± 0.05 mm, inner diameter (ID) 3.4 ± 0.1 mm and a total length of 178 mm was connected to a section of a PEEK high-pressure liquid chromatography (HPLC) column (Applied Research Europe GmbH) with ID 7.5 mm, using TorrSeal epoxy resin (Agilent) (Figure 1A). Larger sapphire tubes (e.g., 10 mm OD) can also be used, provided PEEK HPLC columns with ID ≥ 12.5 mm are available, but such tubes inevitably will have a lower maximal pressure resistance. The 5 mm sapphire tube (Figure 1C(a)) is rated to withstand pressures up to 40 MPa and temperatures surpassing 2000 K. The PEEK HPLC column section with a female capillary connector (Figure 1C(b–c)) was created by sawing a 50 mm HPLC column in half. A series of cylindrical grooves (depth < 0.2 mm) was machined into the inner wall of the capillary connector to provide the adhesive with physical anchoring points next to chemical adhesion. Standard PEEK HPLC parts are rated up to 40 MPa and easily can be used in a temperature range between 173 and 423 K. TorrSeal epoxy resin (Agilent) was selected as adhesive because of its good adhesion to metals, ceramics, and glass, and its resistance to high vacuum forces (Figure S1).^22^ Assembly of the high-pressure column is straightforward and requires no prior experience or know-how. First, the two components of the adhesive (epoxy resin + hardener) are properly dosed and mixed using TorrSeal cartridges and applicator gun.^23^ The sapphire tube is then centered inside the peek HPLC column section using a nitrile rubber O-ring near the open end of the tube (Figure 1C(d)) before carefully filling up the remaining space between the tube and the column with epoxy resin (Figure 1C(e)). An additional O-ring is finally placed around the tube, near the open end of the connector, to ensure perfect vertical alignment of the sapphire tube (Figure 1C(d)), leaving enough space to mount an exchangeable and chemically resistant O-ring (e.g., Kalrez) at the top of the assembly (Figure 1B). Before mounting this last component, the adhesive is allowed to dry for 72 h under a fume hood. The female PEEK end fitting of the HPLC column section (Figure 1C(c)) enables the retrofitted sapphire tube to be connected to a Swagelok gas handling rig using a fingertight PEEK fitting (Figure 1C(h)) and high-pressure PEEK tubing (Figure 1C(i) with a pressure rating of 34.5 MPa). An additional PEEK gasket (Figure 1C(f)) and chemically resistant O-ring (e.g., Kalrez) (Figure 1C(g)) render the connection between the PEEK connector and end fitting leakproof.

### Safety System and Pressure Testing

**Figure 2:**
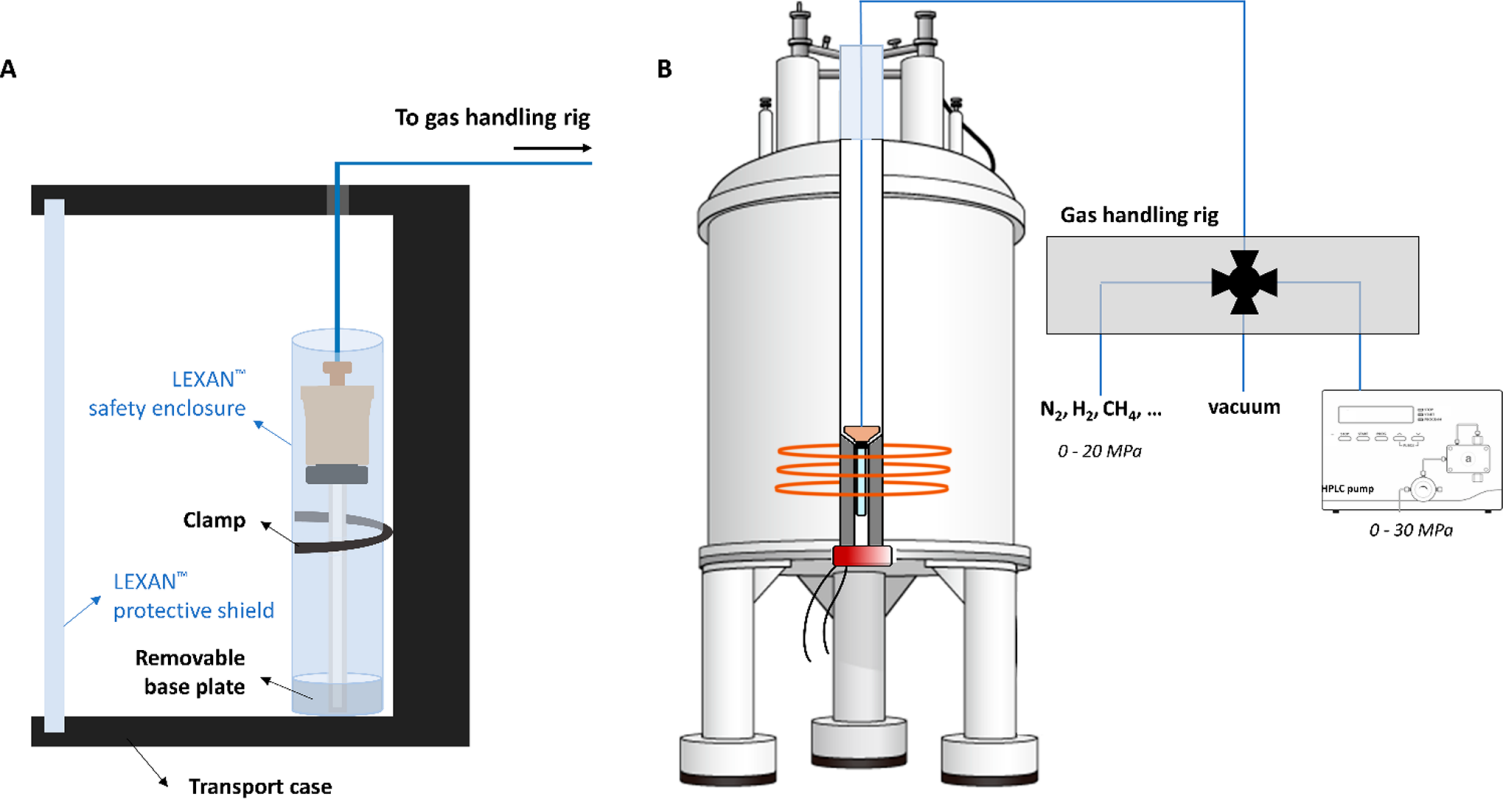
(A) Schematic side-view of the LEXAN safety enclosure and its different components. The enclosure can be clamped onto a case with a protective LEXAN front shield for safe transportation. (B) Removal of the base plate enables one to easily transfer the pressurized tube to the NMR spectrometer without direct exposure. Connection of the cell to a gas handling rig allows pressurization of the sample *in situ* with virtually any fluid.

The fully assembled setup was pressure tested extensively up to 30 MPa using an HPLC pump with water as the fluid. Rigorous pressure testing is mandatory, as one requires a pristine single crystal sapphire tube for construction of this cell. As the quality of the sapphire tubes can depend on the manufacturer and therefore also its pressure resistance can vary between suppliers, rigorous pressure testing of the tube with water pressure while enclosed in the showcased safety enclosure is of critical importance. Subsequent leak testing was performed with combinations of water and N_2_ gas, water and He gas, and water and CH_4_/H_2_ gas over longer periods of time (up to 5 days). A cylindrical safety enclosure with a removable base plate consisting of shatterproof LEXAN allows the pressurized tube to be manipulated outside the magnet, with minimal risk of exposure in the event of hardware malfunction (Figure 2, Video S1). During sample transport and initial pressurization, the tube and cylindrical shield should be secured in an open case with a protective LEXAN front shield. The open ends of the case prevent potentially hazardous pressure buildup, while the LEXAN shields protect the experimentalist against fragmentation. Personal protective equipment including gloves and safety goggles is advised at all times. Photographs of the fully assembled setup and the safety enclosure are shown in Figure S2.

## Experimental Section

### *In Situ*^13^C NMR during THF-CH_4_ Hydrate Formation

**Figure 3:**
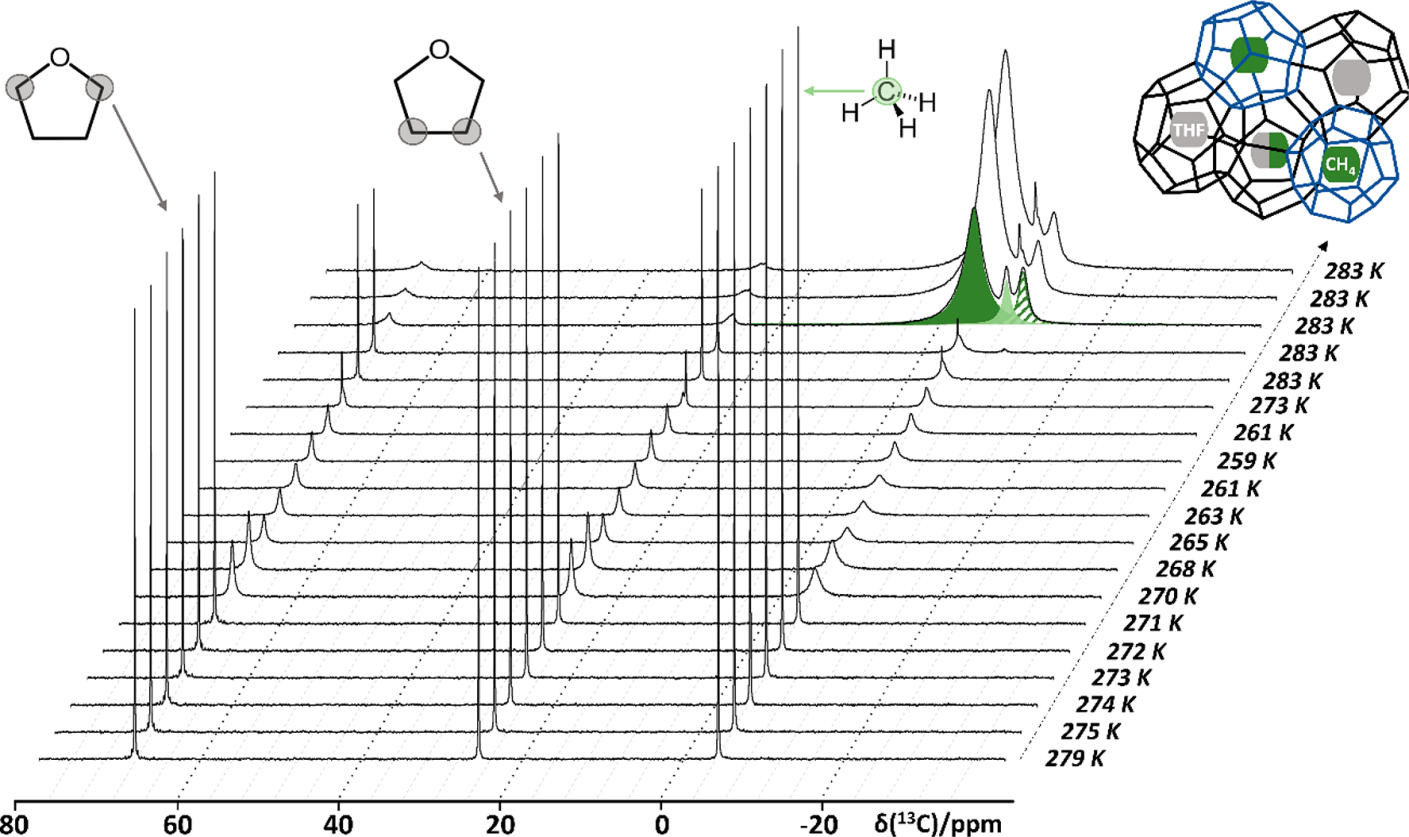
Evolution of the ^13^C NMR spectrum of the D_2_O + THF + CH_4_ system at 6 MPa methane pressure as the physical state of the system evolves from liquid to solid at different temperatures. The system is allowed to equilibrate for 5 min at each temperature before recording the spectrum. The ^13^C resonances originating from THF and CH_4_ are highlighted for clarity. Additional ^13^C resonances emerging near −4.3 (dark green), −8.5 (light green) and −10.4 ppm (dashed green) originate from CH_4_ in the small (blue cages, inset) and large cages (black cages, inset) of the clathrate, along with high-pressure CH_4_ gas percolating in between the porous clathrate hydrate phase, respectively.

The performance of the new high-pressure sample tube was evaluated using NMR spectroscopy to in situ monitor CH_4_ hydrate formation from bulk water at a 6 MPa CH_4_ pressure. Deuterated water (D_2_O, 99.9 atom % D, Merck) was preferred over H_2_O as it allows for easier discrimination between protons originating from CH_4_ and H_2_O. To speed up nucleation and growth of the hydrate, 5.6 mol % of THF (tetrahydrofuran, 99.5%, extra dry over molecular sieves, Fisher Scientific) was added to the water as a kinetic and thermodynamic promoter.^24^ Upon addition of 300 μL of the aqueous THF solution to the sapphire tube, the high-pressure cell was sealed and pressurized to 6 MPa with ^13^C enriched methane gas (CH_4_, Sigma-Aldrich, 99% enrichment) in its safety enclosure. The pressurized tube was subsequently transferred to the NMR magnet. ^13^C NMR experiments were performed using a Bruker Avance Neo 800 MHz spectrometer equipped with a Bruker 5 mm BBO probe head operating at a Larmor frequency of 801.25 MHz for ^1^H and 201.47 MHz for ^13^C. ^13^C NMR spectra were recorded *in situ* while the temperature was varied between 259 and 283 K using a BCU II unit (Figure 3). ^13^C spectra with WALTZ-16 ^1^H decoupling were recorded using a π/2 pulse at 1.88 kHz RF strength and a repetition delay of 60 s.^25^ 32 transients were recorded for each ^13^C spectrum. Chemical shift referencing was performed with respect to TMS, using ethylbenzene (10% in chloroform-d) as the secondary reference with δ(^1^H) = 1.22 ppm and δ(^13^C) = 15.63 ppm (for −CH_3_). The evolution of the ^13^C NMR spectrum of the D_2_O + THF + CH_4_ system at 6 MPa as a function of temperature is shown in Figure 3. Spectral decomposition was performed using DMFit software.^26^

### H_2_/CH_4_ Gas Exchange in Nanoconfined CH_4_ Hydrate

**Figure 4:**
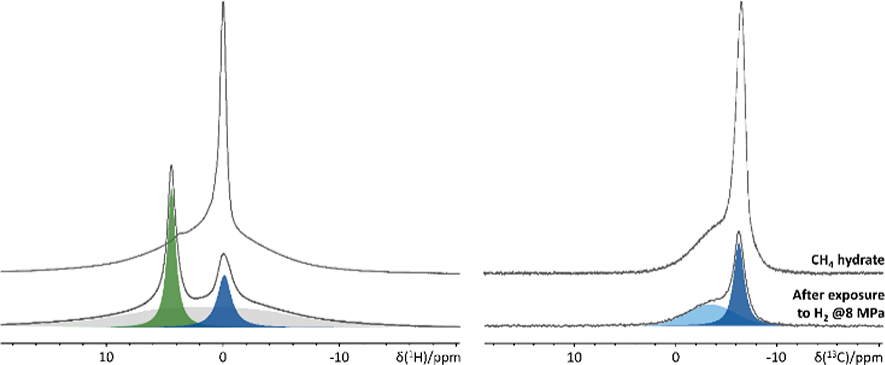
^1^H (left) and ^13^C NMR spectra (right) of the confined CH_4_ deuterohydrate before (top) and after (bottom) exposure to H_2_ gas at 8 MPa for 12 h. The broad resonance in the ^1^H NMR spectrum (highlighted in gray) likely originates from frozen/immobilized residual H_2_O, material background, probe background, etc.

The compatibility of the high-pressure setup with H_2_ gas was tested by evaluating gas exchange of CH_4_ for H_2_ in CH_4_ clathrate nanoconfined in the pores of a reversed-phase silica gel (C_8_-RP, Thermo Scientific Chemicals).^27^ The initial CH_4_ hydrate was formed by packing the bottom section of the tube with 170 mg of C_8_-RP silica gel, adding 112 μL of deuterated water (D_2_O, 99.9 atom % D, Merck), and subsequently sealing and pressurizing the tube with 6 MPa of ^13^C enriched methane gas (CH_4_, Sigma-Aldrich, 99% enrichment). The system was then allowed to react overnight at 261 K. ^1^H and ^13^C NMR spectra of the resulting clathrate hydrate phase were recorded at 261 K (Figure 4, uppermost spectra), using the same instrumentation and the same acquisition parameters for the CH_4_/THF/H_2_O experiment (vide supra). The pressurized tube, containing confined CH_4_ hydrate, was then transferred to an open dewar tube with dry ice (ca. 195 K) and flushed eight times with H_2_ gas to eliminate extraneous methane. Dry ice was used to create a stable, low-temperature environment for flushing the sample without risking damage to the NMR probe or spectrometer in case of hardware malfunction. After the flushing procedure, the cell was transferred back to the NMR spectrometer and the sample was allowed to equilibrate for 12 h at 8 MPa H_2_ pressure at 261 K. Following this equilibration period, ^1^H and ^13^C spectra were recorded in an identical way as described above.

### *In Situ*^1^H MRI of Nanoconfined C_2_H_6_ Hydrate Formation

The formation of ethane hydrate nanoconfined in the pores of C8-grafted reversed-phase silica gel (RPSG, Supelclean LC-8, Merck) was investigated *in situ* using high-field MRI. For this experiment, the bottom section of the high-pressure cell was packed with 150 mg of RPSG material, followed by addition of 90 μL of D_2_O, a volume corresponding to ∼75% of the pore volume. The high-pressure cell was subsequently sealed and pressurized with C_2_H_6_ gas to 3.8 MPa, at 293 K (C_2_H_6_, 99% purity, AGP), avoiding the formation of liquid or supercritical C_2_H_6_. Following pressurization, the system was mounted into the bore of the magnet (21.1 T; NHMFL, Tallahassee, FL, U.S.A.). Experiments were carried out on a 900 MHz spectrometer (Bruker, Avance Neo) controlled with a ParaVision 360 V3.2 (Bruker-Biospin, Boston, U.S.A.), in combination with a modified Bruker Micro2.5 probe with 5 mm coil and imaging gradients having a maximum gradient strength of 1.5 T/m. Upon insertion of the high-pressure sapphire cell, the probe head temperature was lowered to 263 K using an AirJet XR unit (FTS Systems) before starting data acquisition. A spin echo sequence was applied for this purpose by using a repetition time (TR) of 3 s and an echo time (TE) of 5.144 ms. The acquired matrix size was 32 × 32 with a field of view (FOV) of 1.6 × 0.8 cm^2^. Sixteen slices with a slice thickness of 0.5 mm and 0.5 × 0.25 mm^2^ in-plane spatial resolution were averaged over 2 acquisitions. Total acquisition time was 3 min 12 s.

## Results and Discussion

### High-Pressure Tube Design

The newly developed high-pressure sample tube comprises a standard 5 mm single crystal sapphire tube that has been fitted to a section of a relatively inexpensive polyether ether ketone (PEEK) HPLC column. PEEK HPLC tubing and connectors enable integration with a gas rig or a high-pressure pump outside the stray field of the magnet. Adapters to connect the tubing to high-pressure gas connections, e.g., Swagelok, are routinely available from suppliers of HPLC equipment. The cell is compatible with any 5 mm static probe, exhibits an almost zero background in NMR experiments, and allows for use of any liquid, gas, temperature, or pressure range encountered in HPLC experimentation. Built up from standard, inexpensive components, the total material cost of the sample cell easily remains below $1,000, rendering high-pressure MR imaging and spectroscopy widely accessible to investigators in the fields of catalysis, molecular water science, nucleation and crystallization, etc.

### *In Situ*^13^C NMR during THF-CH_4_ Hydrate Formation

Figure 3 shows evolution of the ^1^H and ^13^C NMR spectra recorded *in situ* as a function of temperature during the formation of bulk, binary CH_4_ + THF clathrate hydrate formation. At the start of the experiment, at 279 K, three distinct ^13^C resonances are visible at 68.2, 25.4, and −4.4 ppm. Based on the literature, these signals can be assigned to the two carbon sites in dissolved THF and one in dissolved CH_4_, respectively.^28,29^ Molecular tumbling of the solutes in water averages out intermolecular interactions, resulting in the sharp signals typically observed in liquid-state NMR. As the temperature is lowered to 270 K, water starts to freeze, and the mobility of THF and CH_4_ becomes restricted, broadening the ^13^C resonances of THF and CH_4_. As the temperature is lowered to 259 K, this effect becomes more and more pronounced. Sharp signals only start to reappear once the temperature rises above 273 K and the ice starts to thaw. Interestingly, the D_2_O + THF + CH_4_ system does not return to its initial state. After ±30 min at 283 K, the THF ^13^C resonances have mostly broadened and shifted to 68 and 26 ppm.

At the same time, additional CH_4_ signals at −4.3 (dark green), −8.5 (light green) and −10.4 ppm (dashed green) have emerged (Figure 3) and continued to grow over time. This peculiar behavior points toward the formation of clathrate hydrates occluding both THF and CH_4_ in their cages under the given conditions, more so because of the pressure drop of ±0.4 MPa accompanying this phenomenon.^30^ The observed ^13^C chemical shifts of 68, 26, and −4.3 ppm coincide with THF and CH_4_ contained within the large and small cages of a structure II type hydrate, respectively, which is expected for a THF-stabilized clathrate hydrate.^31,32^ The final ^13^C resonance at −10.4 ppm can be assigned to CH_4_ gas present in interparticle voids and channels created as a result of clathrate hydrate formation.^33^

### H_2_/CH_4_ Gas Exchange in Nanoconfined CH_4_ Hydrate

Figure 4 shows the high-pressure ^1^H and ^13^C NMR spectra recorded upon formation of CH_4_ clathrate hydrate nanoconfined in the pores of C_8_-grafted reversed-phase silica gel (top) and following H_2_/CH_4_ gas exchange (bottom). The occurrence of two resonances at −3.7 and −6.5 ppm in the ^13^C NMR spectrum of the H_2_-exchanged clathrate hydrate points toward a residual fraction of CH_4_ in the small (light blue) and large (dark blue) cages of the structure I clathrate hydrate.^34^ The presence of a signal at −0.15 ppm (dark blue) in the ^1^H NMR spectrum indicates a similar conclusion.^34^ The decreased intensity of these signals upon exposure to H_2_, evident from the comparison in Figure 4, coupled with the emergence of a novel resonance near 4.2 ppm, hints at the successful enclathration of H_2_,^35^ resulting in the formation of a binary H_2_–CH_4_ inclusion complex.

### *In Situ*^1^H MRI of Nanoconfined C_2_H_6_ Hydrate Formation

**Figure 5:**
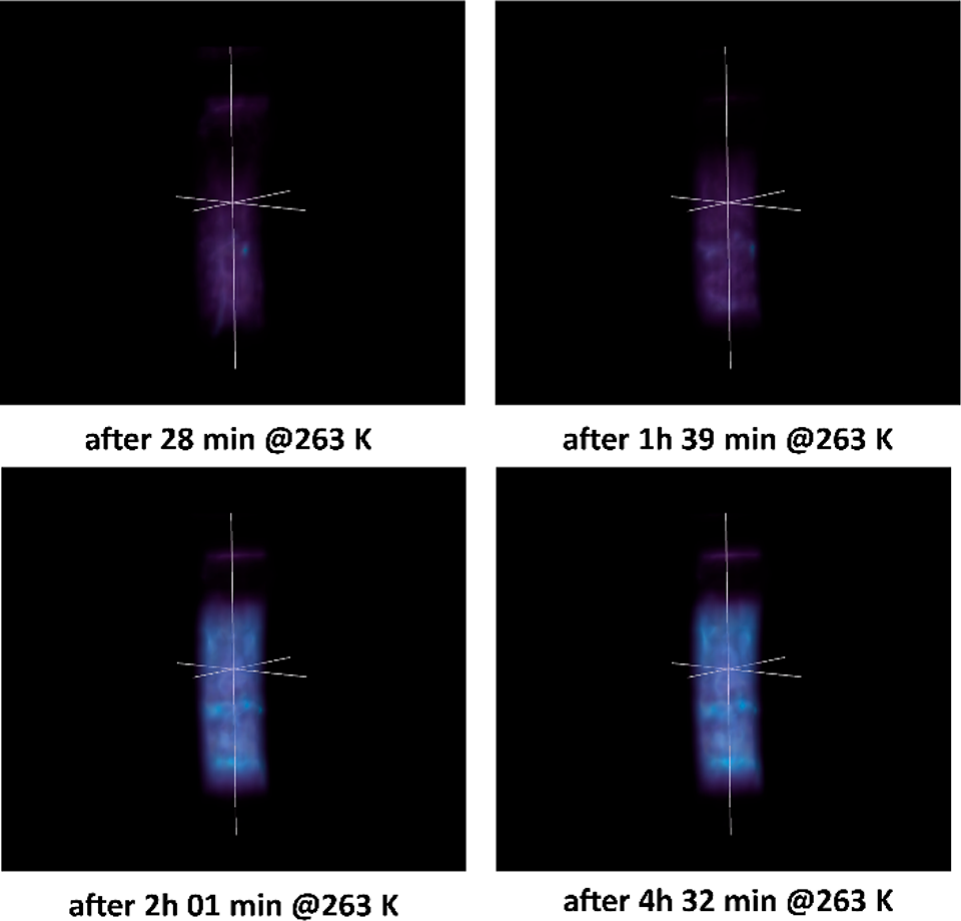
Evolution of ^1^H saturation as a function of time during ethane hydrate formation at 263 K and 3.8 MPa. The start of the measurements, i.e., time = 0 min, coincides with the temperature reaching a steady value of 263 K.

Figure 5 and Figure S3 show ^1^H MRI recorded in situ during the formation of C_2_H_6_ hydrate nanoconfined in the pores of a C_8_-grafted reversed-phase silica gel. The three-dimensional images shown in Figure 5 were rendered in ITK-SNAP (Version 4.0.1) using the built-in 3D rendering module with Gaussian image smoothing (standard deviation = 0.10, approximate max error = 0.03).^36^ In cooling, the system is cooled from room temperature to 263 K and water becomes largely solidified, resulting in low signal intensity in the ^1^H image due to the impeded molecular motion of solid water (Figure 5). Ice-to-hydrate conversion efficiently introduces ethane into the imaged sample volume over time. The growing fraction of mobile ethane molecules incorporated into the cages of the growing nanoconfined clathrate hydrate increases the signal intensity in the ^1^H MRI, resulting in brighter images as a function of increased time.

## Conclusion

The present work documents the development of a low-cost sample environment suited for high-pressure NMR experimentation up to 30 MPa in any commercial 5 mm BBO probe. The sample cell comprises a pressure resistant 5 mm sapphire tube that has been retrofitted with half of a PEEK HPLC column by using a specialized epoxy resin. Connection of the sample cell to standard Swagelok components by means of PEEK tubing enables *in situ* pressurization with virtually any fluid. Using this high-pressure cell, we have been able to monitor the formation of a THF-CH_4_ deuterohydrate at 6 MPa on the minute scale, detailing the evolution of the static ^13^C NMR spectrum of CH_4_ (and THF) as a function of time and temperature. The newly developed setup also enabled a demonstration of the exchange of enclathrated CH_4_ for H_2_ by pressurizing a confined CH_4_ hydrate at 261 K with H_2_ at 8 MPa for 12 h. A third application was also demonstrated, this time in the field of MRI, with the aim of visualizing the enclathration of ethane (C_2_H_6_). Exposure of ice to ethane gas at 3.8 MPa and 263 K results in an increased ^1^H signal distribution over time, showcasing the occlusion of ethane molecules in the cages of the growing clathrate hydrate phase. As they typically form at higher pressures, clathrate hydrates comprise a convenient class of systems for demonstrating the efficacy of a high-pressure cell. The application potential of the newly developed cell is vast and will be of great benefit in many different fields of science, including catalysis and adsorption, that rely on pressure generation and/or stabilization up to 30 MPa, as well as on the concomitant interpretation of the underlying process.

## Supporting Information Available

Additional information on the TorrSeal epoxy resin used to fixate the sapphire tube, photographs of the transport and safety enclosure, and 2D raw data of the MRI experiment (PDF)
Video showing how to handle the high-pressure cell once pressurized (MP4)
