# Supplementary material for: Building a Cost-Efficient High-Pressure Cell for Online High-Field NMR and MRI Using Standard Static Probe Heads: An In Situ Demonstration on Clathrate Hydrate Formation
Source: Anal Chem. 2023 Nov 6;95(46):16936–42. doi: 10.1021/acs.analchem.3c03050 (PMC10666082; doi:10.1021/acs.analchem.3c03050)
Supplement: Supplementary file 1 — ac3c03050_si_001.pdf [file ac3c03050_si_001.pdf]

Supporting Information

Building a cost-efficient high-pressure cell for online high-field NMR and MRI using standard static probe heads: An *in situ* demonstration on clathrate hydrate formation.

Maarten Houlleberghs[1,2], Shannon Helsper[3,5], Dirk Dom[1,2], Thierry Dubroca[5], Bianca Trociewitz[5], Robert W. Schurko[4,5], Sambhu Radhakrishnan[1,2] and Eric Breynaert[1,2,5,6].

[1]NMR/X-Ray Platform for Convergence Research (NMRCoRe), KU Leuven, Leuven 3001, BE

[2]Centre for Surface Chemistry and Catalysis-Characterization and Application Team (COK-kat), Leuven 3001, BE

[3]Biomedical MRI, Department of Imaging & Pathology, KU Leuven, Leuven B-3001, BE

[4]Department of Chemistry and Biochemistry, Florida State University, Tallahassee, FL, 32306, USA

[5]National High Magnetic Field Laboratory, Tallahassee, FL, 32310, USA

[6]Centre for Molecular Water Science (CMWS), 22607 Hamburg, DE

*E-mail: eric.breynaert@kuleuven.be

This supporting information contains additional information on the TorrSeal epoxy resin used to fixate the sapphire NMR tube (S2), photographs of the transport and safety enclosure (S3) and 2D data of the MRI experiment (S3). Video S1 shows how to handle the high-pressure cell once pressurized.

Agilent Data sheet

Torr Seal Low Vapor Pressure Resin Sealant

Torr Seal Mixing System

Technical Specifications

**Absorption**	0.30% (water, 24-hour immersion)
**Acid Resistance**	Withstands SF6 at 25 o C
**Adhesion**	Will not adhere to Teflon, Kel-F, nylon nor polypropylene
**Carcinogenic**	Does not contain any of the carcinogens listed in the OSHA Standards of November 29, 1975
**Color**	Off-white
**Combustion**	(Gasses emitted at normal temperatures) NOx, CO_2, H_2O, CO
**Compressive Strength**	10,000-psi ± 20% (at 25 o C)
**Corrosive Properties**	Corrosive to copper when uncured; non-corrosive when cured
**Cure Time**	24 hours at 25° C, 2 hours at 60° C
**Dielectric Strength**	350 volts/mil
**Dissipation Factor**	0.09 (at 25° C, 1 kHz)
**Expansion, Linear**	30.3 x 10^{-6} in/in/ ° C (at 30° C to 90° C)
**Flash Point**	175° C
**Flexural Strength**	11,000 psi ± 20% (at 25° C)
**Fungus Resistance**	Very high
**Harding Time**	1 to 2 hours at 25° C, 30 minutes at 60° C
**Hardness**	75 - 80 Shore D

Outgassing

See table below for typical sample at various temperatures

Temperature, °C	Cumulative Pumping Time in hrs	Outgassing Rate T-l/cm²/sec
25	1	1.0 x 10^{-5}
25	40	7.5 x 10^{-7}
116 (for 3 hrs)	43	7.0 x 10^{-5}
135 (for 9 hrs)	52	8.0 x 10^{-6}
130 (for 14 hrs)	66	2.0 x 10^{-6}

**Heat of Reaction**	Very slightly exothermic
**Pressure Range**	Suitable for use in pressures of 10^{-9} Torr and below
**Pot Life**	55 minutes (100 grams at 25 o C)

Radiation

Similar epoxy resins have been tested to 10^{11} ergs of gamma radiation without noticeable effect. Torr Seal is a rigid epoxy resin (as opposed to other epoxies which have plasticizers added); all rigid epoxies are relatively impervious to damage from radiation (as compared to other organic materials such as silicone rubber, phenolics and polyesters)

**Resistivity, Volume**	3.52 x 10^{14} ohms/cm (at 25° C)

Shear Strength, Tensile (on an aluminum lap joint)

	at 25 o C, following 7 days at -45 o C, 2150 psi
	at 25 o C, following 7 days at 25 o C, 2000 psi
	at 25 o C, following 7 days at 80 o C, 1900 psi
	at 80 o C, 800 psi
	100% relative humidity, long-term, 1900 psi
	immersed in alcohol, 0.5 hour, 1600 psi
	after 24-hour room temperature cure, 800 psi

**Shelf Life**	12 months minimum from date of shipment from Varian
**Shrinkage, Linear**	0.00125 in/in (at 25° C)
**Specific Gravity**	1.6
**Solvent**	Call Technical Support (800-882-7426)
**Temperature**	-45° C to 120° C; cracks at LN_2 temperatures
**Tensile Strength**	5000-psi ± 30%
**Thermal Conductivity**	10.4 x 10^{-4} cal/sec/cm²/° C/cm
**Toxicity**	Known in cured product - none
**Viscosity**	Thick non-flow paste

Figure S1. Data sheet Torr Seal® low vapor pressure epoxy resin sealant.

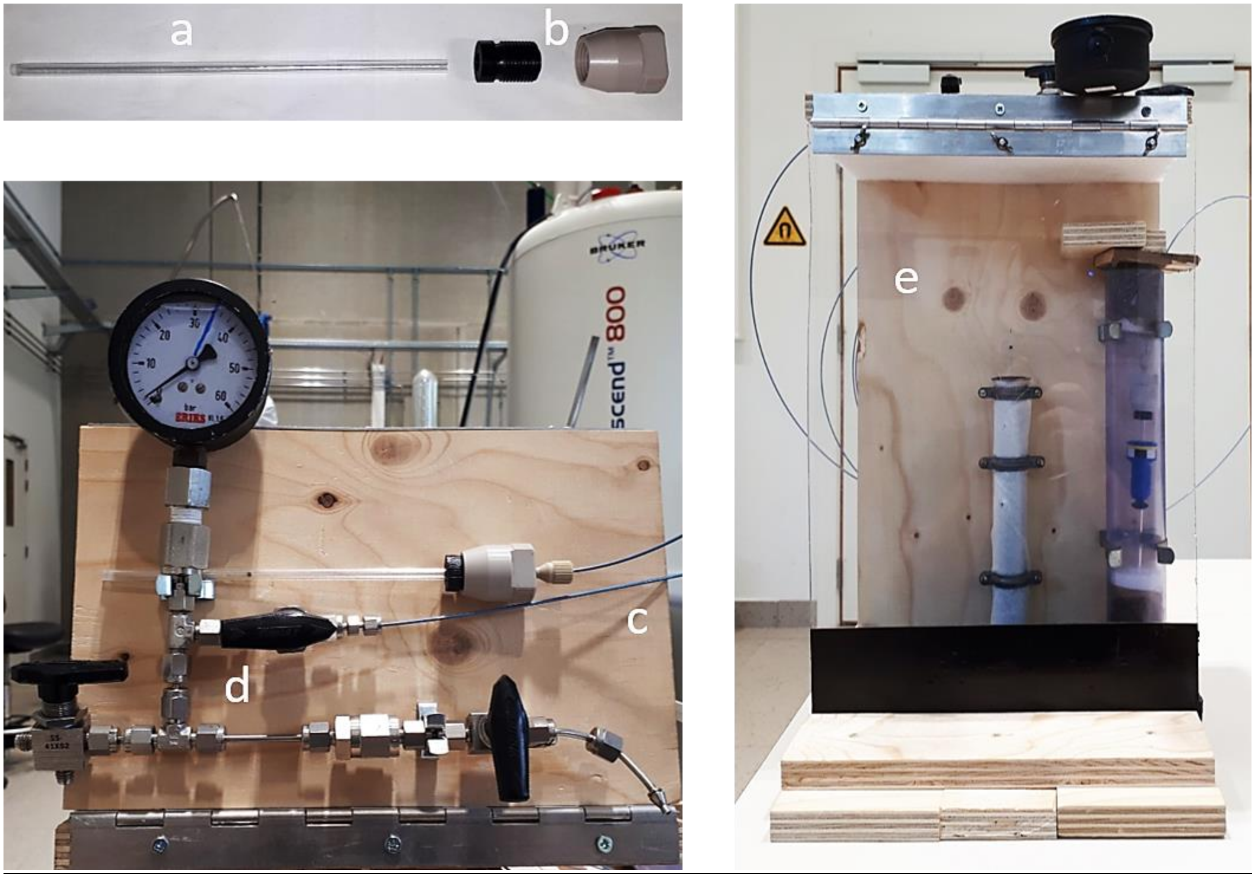

Figure S2. 5 mm sapphire tube (a), connected to a HPLC column (b) through PEEK tubing (c), enabling in situ access to the sample environment with virtually any fluid by means of a built-in gas handling system (d). PMMA-wood based enclosure for safe transportation of pressurized samples.

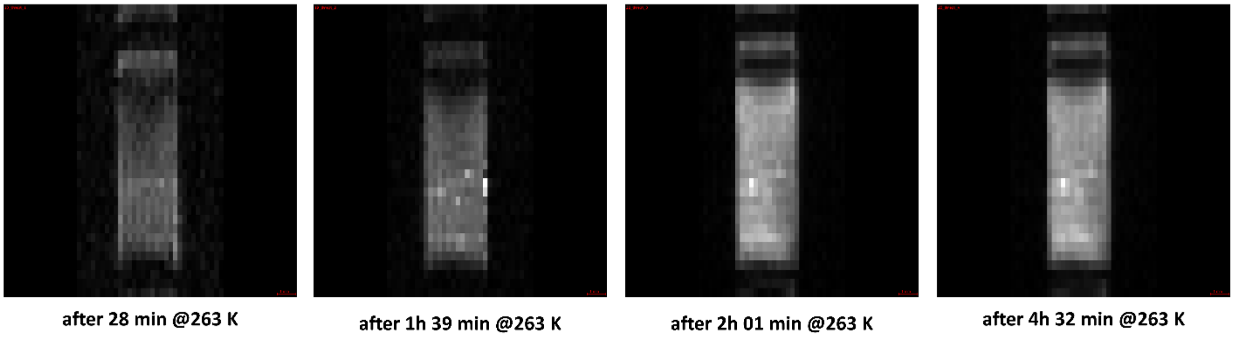

Figure S3. Evolution of ^{1}H saturation in 2D as function of time during ethane hydrate formation at 263 K and 3.8 MPa.
